# Porosity in electrochemical energy storage materials for superior performance gaining: insight into property performance and looking ahead

**DOI:** 10.3389/fchem.2026.1809343

**Published:** 2026-05-13

**Authors:** Pan Shulin, Walid Tahri, Amani Khaskhoussi, Mazen Alshaaer

**Affiliations:** 1 Key Laboratory of Yangtze Aquatic Environment, Research Base, Yibin University, Yibin, China; 2 Department of Engineering, University of Messina, Messina, Italy; 3 Department of Physics, Prince Sattam Bin Abdulaziz University, Al-Kharj, Saudi Arabia

**Keywords:** electrochemical energy storage, pore engineering, porous materials, rechargeable batteries, supercapacitors

## Abstract

The use of renewable energy, electric vehicles, and high-density compact devices requires the power density, long-term stability, and long cycle life of the electrochemical energy storage (ESS) systems. Ion transport, charge storage and mechanical stability of the electrode can be improved with high specific surface area and porous electrodes with well-designed pore structures and high accessibility of electrolytes to the electrodes. The present work critically outlines the current trends in the porous electrode materials, which are porous carbons, inorganic porous structures and coordinated hybrid/composite structures. The relationships between structure, property, and performance that dictate electrochemical behavior and design approaches such as hierarchical porosity, surface functionalization and heterostructure hybridization are pointed out as having a contribution to improving capacitance, energy density and cycling stability. The primary challenges in the manner, in which such materials evolved in the laboratory can be transferred to the practice in the real world, including scaling up to mass production, the volumetric energy density, and the stability of electrodes on the actual cycling, are also stated. Efforts in the recent past have suggested that the objective of pore engineering should be oriented towards equilibrium between the surface area, mechanical stability, and volumetric functionality. The devices of the future EES will need a blend of rational material design and manufacturability approaches to help close the gap between new and innovative porous electrode concepts and commercial energy storage technologies.

## 1 Introduction

Recently, the global trend towards renewable energy, electrically powered cars and the downsizing of electronic equipment has increased the requirement on the development of improved energy storage systems through the electrochemical storage of energy (EES). Such systems are required to provide high energy and power densities, long cycle life, rapid charge/discharge capability and sustained stability to meet such growing demands (Ran et al., 2024). On the other hand, traditional electrode materials are not typically meet the criteria as they have small active surface area, slow ion transport kinetics, and structural instability at high-rate and rapid charging (Hu et al., 2025). Consequently, the next-generation EES Systems have become central to research based on porous materials with high specific surface area, controllable pore arrangement, and increased electrode-electrolyte interfaces (Li H. J. et al., 2024; Yu et al., 2025). The concept of pore engineering is now a standard practice in high-performance electrode material design because the recent breakthroughs in the field of electrochemical energy storage have revealed that pore confinement at sub-nanometer scales can significantly enhance the electrochemical capacitance in a way that is not predicted by classical models of electrochemical reaction between two polymer layers (Guo et al., 2024; Zhang X. R. et al., 2024). Such new results accord with classic earlier investigations showing that sub-nanometer pore confinement is able to produce an anomalously large capacitance enhancement than that where only two layers of material are involved and as such prove pore-size matching to be a general principle in porous electrode design.

In general, porous architectures have brought many active sites, effective charge storage, and enhanced ion mobility, and thereby enhance electrochemical functions within a range of applications, such as the supercapacitors, rechargeable batteries, and new hybrid systems (Liu Y. et al., 2022; Ran et al., 2024). Notably, the hierarchical pore structure comprising of micropores, mesopores, and macropores is quite efficient in maximizing surface area, mass transportation, reducing mechanical stresses during cycling, and providing high-rate performance and stability of the cycle (Gao et al., 2020; Wu et al., 2025; Zhang and Xie, 2025). Accordingly, the interrelationship between the structure, properties and performance of porous materials is fundamental to the optimization of the electrode behavior to particular applications and such has been fully investigated in recent work on porous carbons, inorganic porous electrodes and framework-derived hybrids (Cui et al., 2025; Hu et al., 2026; Yu et al., 2018; Fan et al., 2026).

Therefore, this brief review brings together new developments in porous materials towards high-performance electrochemical energy storage with particular emphasis on the role of pore architecture in electrochemical performance. Mainly, the main categories of materials including porous carbons, porous inorganic materials, and hybrid or framework-based systems are examined in terms of their application in supercapacitors, rechargeable batteries, and energy devices in the future. What is more, major issues and future trends of the rational pore engineering and its practical implementation are also pointed out. In consequence, porous materials with highly developed and ideal pore structures have been relied on in supercapacitors, batteries, and hybrid devices to store electrochemical energy more efficiently. This is due to their enhancement of surface area and structural integrity, as well as ease of ion movement. In larger scale, this work highlights the importance of matching pore hierarchy with electrochemical measures instead of just using values of the surface area. Still, differences of performance comparisons across studies indicate the necessity of standardization evaluation protocols. By filling these gaps, it will be possible to have more definite structure-performance benchmarks and to hasten the rational design of next-generation porous electrodes. Accordingly, a special concern is taken to the combined role of pore-size hierarchy, surface chemistry, and structure design to determine electrochemical behavior, focusing on quantitative trends and classical works that defined the basic structure-property-performance relationships in porous electrodes.

## 2 Porous material classes for EES

### 2.1 Porous carbon materials

Among various materials, the porous carbons with their inherent electrical conductivity, large specific surface area, and chemical stability are one of the most widely studied classes of electrode materials in electrochemical systems applications (Sayed et al., 2024; Zhang Z. et al., 2024; Zhang and Xie, 2025). Specifically, activated carbons, carbon aerogels, graphene derivatives, and heteroatom-doped carbon nanostructures fall in this category. As a result, porous carbons have greater capacitive charge storage ability and rapid ion conduction, which makes them especially ideal in supercapacitors and battery electrode applications (Pal et al. 2023; Peng et al., 2024).

In detail, activated carbons contain micro- and mesopores that give it a high concentration of active sites and allow fast ion transport routes. Accordingly, recent studies show that hierarchical carbon spheres including the micropore and mesopore structures have enhanced capacitance performance because of the ease of electrolyte to reach the inner surfaces of the structure (Chen et al., 2024; Nayak et al., 2024; Wang et al., 2022). Moreover, doping of porous carbons with heteroatomic (e.g., N, O, S) enhances the surface wetting and adds pseudocapacitive effects to the total charge storage capacity, thus increasing the storage capacity of porous carbons multi-folds with heteroatomic doping of carbons (Wang et al., 2019; Wang et al., 2022; Wu et al., 2021).

In addition, MOF-based porous carbon materials have gained a lot of interest due to the pore environment which can be customized and the high pore surface area that they possess. Correspondingly, MOF-based carbon structures have been shown to have promising behavior of use in supercapacitors and battery electrodes, due to their hierarchical pore structure and increased electronic conductivity (Lobato-Peralta et al., 2024; Yu et al., 2019).

On the whole, porous carbons (activated, doped, or MOF-derived) show definite capacitances of 150–500 F·g^-1^, and cycle retention often over 90 per cent, to demonstrate the effect of hierarchical porosity and heteroatom doping on ion accessibility, conductivity and charge storage performance (Table 1).

**TABLE 1:** EES storage devices utilizing representative porous materials, the structure-performance relationships of these materials.

Material type	Examples	Characteristics of the pore and its structure	Role of dominant pore size	Emphasis on synthesis	Electrochemical performance	Applications	Ref
Porous Carbons	Activated carbon, Graphene, CNTs	SSA: 500–3000 m^2^/g; micropores 0.5–2 nm, mesopores 2–50 nm	Micropores govern charge accumulation, whereas mesopores enable ion diffusion	Physical/chemical activation, templating	Specific capacitance: 150–350 F/g; cycle retention >90%	Supercapacitors, hybrid capacitors	Sayed et al. (2024), Zhang D. et al. (2024), Zhang and Xie (2025)
Heteroatom-Doped Carbons	N-doped, O-doped, N,O-co-doped carbons	SSA: 800–2500 m^2^/g; micropores/mesopores; surface functional groups	Micropores increase capacitance, while mesopores enhance electrolyte accessibility	Doping via ammonia, urea, or melamine; pyrolysis	Capacitance: 200–450 F/g; improved rate capability; pseudo-capacitance contribution	Supercapacitors, Li/Na-ion batteries	Chen et al. (2024), Lobato-Peralta et al. (2024), Nayak et al. (2024), Wang et al. (2019), Wang et al. (2022), Wu et al. (2021), Yu et al. (2019)
MOF-Derived Porous Carbons	ZIF-8, ZIF-67, UiO-66 derivatives	SSA: 1000–3500 m^2^/g; micropores and mesopores; hierarchical porosity	Hierarchical micro/mesopores strengthen storage and rate capability	MOF pyrolysis under N2; controlled carbonization	Capacitance: 250–500 F/g; cycle retention >95%; high-rate performance	Supercapacitors, Li-ion, Na-ion batteries, hybrid devices	Chen et al. (2023), Guo et al. (2022), Li X. et al. (2023), Mahajan et al. (2024), Ramasubbu et al. (2020), Rangaraj et al. (2023), Wang and Zhang (2023), Yang et al. (2025), Zhang et al. (2023), Zhao et al. (2026), Zhu T. et al. (2023), Zhu B. et al. (2024)
COF-Derived Porous Carbons	TpPa-1, TpBD, 2D/3D COFs	SSA: 800–2200 m^2^/g; uniform mesopores; high structural order	Uniform mesopores support rapid ion transport and stable charge propagation	Pyrolysis of COFs; post-synthetic doping possible	Capacitance: 200–420 F/g; cycle retention >90%; flexible electrode performance	Flexible supercapacitors, Li-ion batteries	Wan et al. (2024)
Porous Inorganic Materials	Mesoporous TiO_2_, MnO_2_, NiO, V_2_O_5_	SSA: 80–300 m^2^/g; mesopores 2–20 nm; crystalline frameworks	Mesopores promote redox accessibility and ion transport	Sol-gel, hydrothermal, template-assisted	Pseudocapacitance: 150–350 F/g; fast redox kinetics	Pseudocapacitors, Li-ion batteries	Li H. et al. (2023), Ramasubbu et al. (2020) , Zhu G. et al. (2024)
Hybrid/Framework-Derived Systems	MOF-carbon composites, MOF-metal oxide	SSA: 500–2500 m^2^/g; hierarchical pores; conductive network	Hierarchical pores enhance the power-energy balance	MOF pyrolysis with metal oxide incorporation	Capacitance: 300–550 F/g; cycle retention >95%; high energy and power density	Supercapacitors, hybrid devices, batteries	Jang et al. (2023)
Biomass-Derived Porous Carbons	Coconut shell, Rice husk, Sawdust	SSA: 700–2000 m^2^/g; micro/mesopores; sustainable	Micro/mesoporous interaction generates sustained capacitance	Carbonization and chemical activation (KOH, ZnCl_2_)	Capacitance: 150–350 F/g; eco-friendly, scalable	Supercapacitors, Na-ion batteries	Sun et al. (2022), Wang et al. (2022)
Hierarchical Porous Electrodes	Carbon + metal oxide composites	Macro/meso/micropore integration; stress accommodation	Macro/meso/micropores reduce transport resistance and stress	Templating, composite synthesis, solvothermal	Capacitance: 300–500 F/g; cycle retention 90%–98%; enhanced ion transport	Hybrid capacitors, batteries	Du et al. (2024), Wang et al. (2023), Zhang Z. et al. (2024)
3D Porous Carbon Frameworks	Aerogels, foams, graphene sponges	SSA: 600–2500 m^2^/g; interconnected pores; flexible structures	Interconnected meso/macropores improve electrolyte flow	Freeze-drying, hydrothermal assembly	Capacitance: 250–400 F/g; high-rate stability; mechanical flexibility	Flexible supercapacitors, wearable devices	Fan et al. (2015), Zheng et al. (2024), Zhu et al. (2024a)
Hierarchical MOF/COF Hybrids	ZIF-8@TiO_2_, COF@metal oxide	Micro-mesoporous structure; hybrid interfaces	Micro-mesoporous surfaces enhance multipath transport	MOF/COF templating with metal oxides	Capacitance: 350–500 F/g; cycle retention >95%; high energy density	High-power supercapacitors, Li-ion batteries	Kitchamsetti and Kim (2023) Li et al. (2024a), Mahajan et al. (2024), Zhou et al. (2024)

Although they possess good electrochemical properties, porous carbons continue to have the problem associated with low volumetric energy density due to overly large porosity. Moreover, the stability of doped systems over harsh cycling conditions is not well-known. The next-generation efforts ought to thus be directed towards pore densification, controlled heteroatom placement and scalable chemistry to achieve the highest possible levels of gravimetric and volumetric performance.

### 2.2 Porous inorganic materials

In contrast to carbon-based systems, the utilization of organic substances as faradaic energy storage inorganic binary materials, including transition metal oxides, sulfides, phosphides, and nitrides, are important because of their variety of oxidation states and redox active surfaces (Liu X. et al., 2022; Mo et al., 2023; Sun et al., 2022). When designed appropriately, these materials are designed to facilitate fast ion transport and eliminate stress at the multiple charge-discharge cycles when designed with porous architecture. For instance, hierarchically porous TiO_2_ and MnO_2_ structures have already been a widely studied anode and pseudocapacitive material due to their porous structures that allow the free flow of electrolytes and increase redox activity on the surface. Furthermore, three-dimensional, ordered porous inorganic structures have been studied on the basis of outstanding rate performance and stability (Li H. et al., 2023; Ramasubbu et al., 2020; Zhu B. et al., 2024). Importantly, hierarchical porosity is important in accelerating diffusion rates and encouraging the rate performances of the ion diffusion process through the porous inorganic electrodes like mesoporous TiO_2_ and MnO_2_, which consequently demonstrated rapid redox kinetics and pseudo-capacitance values of 150–350 F·g^-1^ (Table 1).

Despite having potentially high theoretical capacity, porous inorganic materials have poor intrinsic conductivity and structural degradation during cycling. This shows the significance of bonding organic phases with the conductive structures or carbonaceous matrices. Hybridization and interface engineering will be the main focus towards stabilizing redox-active sites and maintaining rapid ion transport in the future.

### 2.3 Hybrid and framework-derived porous materials

More recently, MOF-derived and COF-derived carbons and other porous materials based on frameworks combine the advantages of high porosity with desired chemical functionality and functionality (Chen et al., 2023; Guo et al., 2022; Wang and Zhang, 2023; Yang Y. et al., 2024). In this regard, MOFs are crystalline porous materials with adjustable pore sizes and characteristics, which makes them an appealing target of hybrid electrode materials (Mahajan et al., 2024; Zhang et al., 2023). Further, carbonization of MOFs or composing conductive phases like graphene, carbon nanotubes (CNTs), or metal oxides into the frameworks of MOFs has allowed researchers to produce porous hybrid systems with increased conductivity and electrochemical activity. As a result, these hybrids combine faradaic and capacitive storage properties, which leads to a higher energy and power performance of the electrodes (Rangaraj et al., 2023; Yang et al., 2025; Zhu T. et al., 2023; Xu et al., 2015). Similarly, COF generated porous carbons create a route to crystalline, organized networks of pores, which may increase ion transportation and cycling stability (Li X. et al., 2023; Zhu T. et al., 2023).

Overall, hierarchical pores in combination with conductive networks combine capacitive and faradaic processes to produce high-energy and power electrodes. Consequently, MOF/COF composites, which are hybrid and framework-derived porous materials, have reached capacitances of 300–550 F·g^-1^ with greater than 95 per cent cycle retention as seen in Table 1. On the other hand, the structural failure of structures in the process of thermal conversion and the expensive nature of precursor synthesis are still major challenges towards commercialization. Moreover, there is no accurate control of pore development during carbonization. The solution to these problems will involve low-temperature synthetic pathways, characterization *in situ* and design of frameworks that are porous when large-scale processed.

## 3 Structure, property, performance relationships

Basically, the functionality of porous electrodes is inherently associated with pore structure, surface chemistry and tissue connectivity between the structure and pore space structure (Jiang et al., 2024; Peng et al., 2024). Specifically, micropores (less than 2 nm) give many active sites on which the charge can be stored although it may hinder the rapid transportation of ions at high rates. By comparison, mesopores (2–50 nm) and macropores (higher than 50 nm) act as ionic pathways reducing the resistance to diffusion and increasing the rate capacity. Thus, orderly arrangement of different types of pores results in enhanced penetration of electrolytes, effective ion transport channels, and reduced transport resistance (Resing et al., 2025; Yang K. et al., 2024; Zhu B. et al., 2024). Such a structure-dependent electrochemical behavior has continued to be established in early work. These investigations concluded that the best electrochemical performance is achieved when ion diffusion impedance is as low as possible in the mesopores and charge accumulation in the micropores is as large as possible, resulting in the fundamental structure-property-performance correlation in porous electrodes (Guo et al., 2024; Zhang X. R. et al., 2024).

In the same way, the electrode structures of batteries with multiscale pore architecture enable high-rate charging through reducing the concentration gradient and uniform distribution of ion flux (Wang et al., 2025; Zhao et al., 2026). Additionally, porosity also affects stability in a cycle where volumetric changes are accommodated due to the insertion and extraction of ions especially in alloying type or conversion type electrodes (Guo et al., 2024; He et al., 2025; Zhang X. R. et al., 2024).

Recently, quantitative relationships between pore size distribution and electrochemical performance measures were also found recently. For example, sub-nanometer micropores (around 0.5–1.2 nm) have the potential to increase the ratio of double-layer capacitance by almost 20–40 percent with respect to the ion packing and partial desolvation effects within confined nanopores (Li et al., 2025; Qing et al., 2021). Mesopores in the 5–20 nm range are known to lower the resistance to ion diffusion by factors of 30–60 over purely microporous structures and can enhance rate capability by a similar amount. Macropores (>50 nm), in turn, are primarily electrolyte reservoirs by which electrolyte rapid penetration and reduction of transport impedance occur but which have comparatively small contributions to capacitance. As such, hierarchical systems that combine micro-, meso-, and macropores have been identified as the most desirable systems in terms of balancing charge storage, ion transport, and long-term stability of electrochemical processes in energy storage systems (Du et al., 2024; Du et al., 2026; Fan et al., 2015; Jang et al., 2023; Kitchamsetti and Kim, 2023; Li S. et al., 2024; Wan et al., 2024; Wang et al., 2023; Wei et al., 2025; Zhang D. et al., 2024; Zhang Y. et al., 2024; Zheng et al., 2024; Zhou et al., 2024).

**FIGURE 1:**
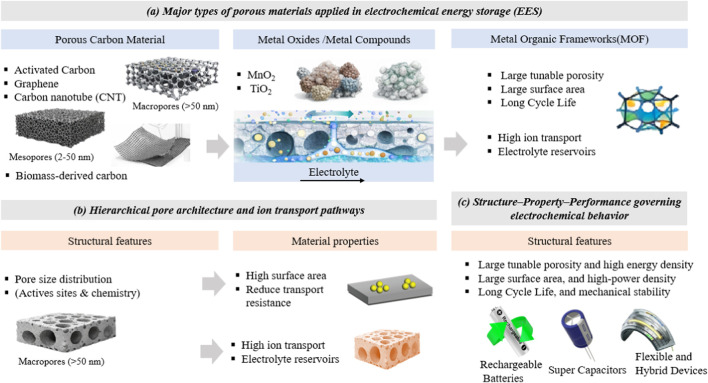
Electrochemical energy storage schematic: **(a)**. key classes of porous materials applied in EES, **(b)** hierarchical pore structure and ion transport pathways, and **(c)** structure-property-performance relationship that dictates the electrochemical behavior.

Finally, Figure 1 shows the functionalization of surfaces, micropores, mesopores, and macropores in determining electrochemical performance to establish a schematic description of the relationship among structure, properties, and performance. In another word, the figure presents three main parts: (a) key classes of porous materials that provide a high surface area and accessible active sites to facilitate electrochemical reactions, (b) hierarchical pore structure and ion transport pathways where micropores, mesopores and macropores aid efficient ion movement, access and storage of electrolytes and charges, and (c) structure-property-performance relationship that dictates the electrochemical behavior by enabling ion diffusion, accessibility of electrolytes, charge storage.

On the contrary, supercapacitors, lithium-ion and sodium-ion batteries and hybrid systems are examples of porous materials and their structure-performance relationships are identified in Table 1. To further reinforce the structure property performance analysis, Table 1 displays exemplary porous electrode material which has been reported in recent reports with focus on the pore properties and functional roles of pore sizes, electrochemical performance, and applicability. The comparative summary proves that the electrochemical optimization process is based on a balance between the micropore-enhanced charge storage and mesopore-enhanced ion transport and macropore-enhanced ion accessibility.

As indicated in Table 1, data show that there are various trends: first, ion transport and rate performance increase with hierarchical pore topologies that are integrated with micro-, meso-, and macropores. In parallel, MOFs and COFs have a high surface area and tunable functionalities that can be used to improve capacitance and cycling stability. Another important strategy, the second approach of enhancing electrode-electrolyte interface is through the application of the hybrid or doped materials. Appropriately, engaging in advanced approaches to energy storage in electrochemical systems requires designing porous materials that balance aspects of energy density, power density and stability at longer times. Collectively, these results demonstrate that it is possible to achieve this by rational pore engineering and functionalization. Likewise, hierarchical pore structures are closely linked to an improved electrochemical performance: in this case, ionic pathways are optimized between micro, meso, and macro pores and, as a result, capacitance and cycling stability is improved across supercapacitors and batteries that utilize carbons based on MOFs and COFs with high specific surface areas (>1000 m^2^ g ^-1^) as indicated in Table 1.

Although the benefits of hierarchical porosity are quite obvious, the establishment of the most efficient balance between micropore-based charge storage and meso/macropore-based ion transfer is one of the primary design concerns. Besides, electrolyte chemistry of the surface functional group interacts decisively with both long-term cycling stability and rate performance. Future studies must thus combine the pore multiscale modeling in the context of situ electrochemical characterization to develop quantitative structure performance relationship.

## 4 Applications in high-performance EES systems

### 4.1 Supercapacitors

Essentially, the porous material is one of the essential elements of performance of the supercapacitors since high surface areas increases not only the double-layered capacitance but also the pseudocapacitive reactions (Du et al., 2024; Du et al., 2026; Fan et al., 2015; Jang et al., 2023; Qiu et al., 2023; Wan et al., 2024; Wang et al., 2023; Wei et al., 2025; Zhang D. et al., 2024; Zhang Y. et al., 2024; Zhang and Xie, 2025; Zhu et al., 2024b). In particular, Carbon-based electrodes with intricate pore structures provide high specific capacitances and high performance in symmetric and asymmetric devices. Then, pseudo-capacitance and conductive composite structures are further enhanced when heteroatoms and conductive structures are introduced to improve the device durability (Li H. J. et al., 2024; Ma et al., 2025). As summarized in Table 1, indicates that electrodes prepared using hierarchical porous carbons and hybrid composites can be prepared to achieve specific capacitances of up to 500 F·g^-1^ and at the same time have high-rate capability. This observation supports the fact that the association of micropores, acting as active sites, with meso- and macropores, which help to carry out quick ion transportation, is especially effective. However, microporosity can be too large at high current densities and cause reduced power output at fast charge discharge conditions. Hence, optimization of the pore size distribution as opposed to maximization of the surface area by itself is important in practical operation of supercapacitors. Hierarchical architectures and stable surface functionalization should be the future focus of supercapacitor designs as these will improve both energy efficiency and long-term durability.

### 4.2 Rechargeable batteries

Likewise, the so-called porous electrodes have a significant impact on the results of lithium-ion, sodium-ion, and the promising battery technology by improving ion diffusion, electrode polarization, and mechanical stresses (Li et al., 2024b; Zhang Q. et al., 2024; Zhu et al., 2025). Notably, MOFs used as a source of porous carbons have very high lithium storage capacities, highlighting the significance of porosity in the design of a battery (Chu et al., 2024; Zhu B. et al., 2024). In addition, hierarchically porous anodes that are developed to facilitate a quick charge transfer showed enhanced kinetics and lowered overpotential due to the creation of effective ion pathway by multiple length scales (Naumann et al., 2023; Zhang X. R. et al., 2024; Zhu G. et al., 2023). As shown in Table 1, the electrodes made of MOF-derived porous carbons can improve the rate performance and exhibit extremely high lithium/sodium storage capacities, thereby further prove that hierarchical porosity enhances energy density and cycling stability in real batteries. In spite of these benefits, the battery electrodes with high porosity are prone to lower volumetric capacity, and densification of electrodes. Moreover, cyclic volume expansion and contraction can cause mechanical degradation even with prolonged use as it happens. These constraints will need more sophisticated binder systems, pore-reinforced architectures, and electrode compaction schemes which can be used in commercial cell format.

### 4.3 Emerging and hybrid systems

Beyond traditional batteries and supercapacitors, porous materials also play a role in creating new energy storage schemes, such as metal-ion capacitors, flexible, and hybrid faradaic-capacitive technologies (Catarineu et al., 2023; Kitchamsetti and Kim, 2023; Li S. et al., 2024; Qiu et al., 2023; Sun et al., 2024; Zheng et al., 2024; Zheng et al., 2025; Zhou et al., 2024). In these systems, such applications often require very specific porosity and hybrid interfaces to achieve desired energy and power requirements. As evidenced in Table 1, customized pore structures are useful in the balance of energy and power in improved electrochemical devices; new metal-ion capacitors and hybrid systems reach capacitances of 350–500 F·g^-1^ with cycle retention of more than 95 percent through the use of hierarchical and hybrid porous structures.

On the other hand, this makes the problems of charge storage in hybrid systems complicated and the mechanisms are difficult to predict precisely in performance and optimize the devices. Interfacial compatibility between capacitive and faradaic elements is also an issue of serious concern especially in the case of flexible or solid-state applications. The research in the future must be based on interface-engineering, scalable-fabrication, and real-time-diagnostics images that will unlock the potential of new hybrid-energy storage methods.

## 5 Challenges and opportunities

Currently, despite significant progress in porous electrode materials, there are still numerous challenges (Ju et al., 2023; Li et al., 2024a; Wan et al., 2024).

- Regarding structural stability Although high-current-density operation and long-cycle operation require the material to maintain its integrity, it is necessary to develop a material with new properties;
- With respect to volumetric energy density, large surface area tends to reduce packing density, which limits the energy per unit volume;
- Concerning scalable synthesis There is a need to have a material with new properties, but these materials require cost-effective, reproducible, and environmentally friendly fabric The development of new synthesis routes, especially to be used in large-scale industrial processes, can and should take into account cost-effectiveness and the environmental performance;
- Accordingly, the development of new synthesis routes, especially to be used in large-scale industrial processes, can and should take into account cost-effectiveness and the environmental performance (Kang and Kim, 2024; Oh et al., 2025; Sun et al., 2025).

Beyond that, the combination of multiscale modeling and experimental synthesis may be employed to predict the behavior of materials in actual operating conditions with minimal use of the trial-and-error methods. The use of high-level characterization tools in conjunction with machine learning will increase the identification of strong porous structures. Lastly, these materials will need to be scaled up to industrial production through modular, scalable, and environmentally friendly synthesis methods that will be necessary to design.

## 6 Conclusion and future perspectives

Overall, high-performance electrochemical energy storage (EES) systems are now unthinkable without porous materials that help them to increase ion conductivity, offer high concentration sites, and allow structure change in repeated cycling. Advanced pore engineering solutions increasingly concentrate on quantitative matching of pore hierarchy and electrochemical requirements rather than just maximizing particular surface area, demonstrating the evolution of design principles established by key and contemporary high-impact studies. Specifically, micropores that hybridize with mesopores and macropores can lead to a balance between the energy density, power density, and cycling stability of supercapacitors, lithium/sodium-ion batteries, and more sophisticated hybrid devices. Among available options, porous carbons, inorganic porous structures, and hybrids of MOFs and covalent organic frameworks (COFs) among the variety of other materials are of particular interest in terms of tunable porosity, large surface area, and chemical functionality.

Nevertheless, despite these advances, there are major challenges, such as structural integrity under rapid charging and high cycle rates, high volumetric energy densities without impairment in porosity and scalable sustainable manufacturing technology. Further, the incorporation of porous electrodes in developed flexible, solid-state or hybrid systems requires careful design of the electrode-electrolyte-interfaces, the connectivity of the pore and mechanical strength.

Looking ahead, the subsequent studies need to focus on rational pore engineering and multiscale optimization concepts. Potentially, solutions that have been suggested like self-healing porous frameworks, hybrid electrodes based on capacitive and faradaic processes, environmentally friendly fabrication methods and *in situ* experimental studies of ion transport and structural development are very promising when it comes to actual applications of energy storage. Provided that synthesis and integration of devices proceed, it is possible that within the next five to 10 years, the mass production of these materials in flexible and wearable energy storage devices can become a reality due to continued attempts to industrialize them. Ultimately, porous materials are therefore highly adaptable and versatile solutions to the ever-increasing energy storage needs of renewable energy systems, electric vehicles, and portable electronics. With continued advances in pore structure, material chemistry and device coupling, they are expected to serve as the next-generation of high-performance energy storage systems.

To conclude, it can be anticipated that the next-generation of EES systems of high performance will be enabled by the integration of computational design, hierarchical pore engineering, and sustainable synthesis. Also, flexible or solid-state platforms can be combined with optimized porous materials to open up to wearable and portable electronics. Lastly, the desire to have interdisciplinary approaches between materials chemistry, device engineering, and environmental sustainability will play a significant role in the implementation of viable, scalable and long-term energy storage solutions.
